# Test results comparison and sample stability study: is the BD Barricor tube a suitable replacement for the BD RST tube?

**DOI:** 10.11613/BM.2020.030704

**Published:** 2020-10-15

**Authors:** Sanja Mandić, Dario Mandić, Iva Lukić, Tara Rolić, Vesna Horvat, Maja Lukić, Silvija Osvald, Vatroslav Šerić

**Affiliations:** 1 Institute of Clinical Laboratory Diagnostics, Osijek University Hospital, Osijek, Croatia; 2 Department of Chemistry, Biochemistry and Clinical Chemistry, Faculty of Medicine, University of Osijek, Osijek, Croatia

**Keywords:** BD Barricor tube, stability, serum, plasma, evaluation

## Abstract

**Introduction:**

The aim was to evaluate the BD Barricor tubes by comparison with the BD Rapid Serum Tubes (RST) through measuring 25 analytes and monitoring sample stability after 24 hours and 7 days.

**Materials and methods:**

Samples of 52 patients from different hospital departments were examined. Blood was collected in BD RST and BD Barricor tubes (Becton, Dickinson and Company, Franklin Lakes, USA). Analytes were measured by Beckman Coulter AU 480 (Beckman Coulter, Brea, USA), Dimension EXL (Siemens Healthcare Diagnostics, Newark, USA) and ARCHITECT i2000SR (Abbott Diagnostics, Lake Forest, USA). Between-tube comparison for each analyte was performed, along with testing analyte stability after storing samples at 4 °C.

**Results:**

BD Barricor tubes showed unacceptable bias compared to BD RST tubes for potassium (K) (- 4.5%) and total protein (4.4%). Analyte stability after 24 hours was acceptable in both tested tubes for most of analytes, except for glucose, aspartate aminotransferase (AST) and lactate dehydrogenase (LD) in BD Barricor and free triiodothyronine in BD RST sample tubes. Analyte stability after 7 days was unacceptable for sodium, K, calcium, creatine kinase isoenzyme MB, AST, LD and troponin I in both samples; additionally for glucose, alkaline phosphatase and albumin in BD Barricor.

**Conclusion:**

All analytes, except K and total protein, can be measured interchangeably in BD RST and BD Barricor tubes, applying the same reference intervals. For most of the analytes, sample re-analysis can be performed in both tubes after 24 hours and 7 days, although BD RST tubes show better 7-day analytes stability over BD Barricor tubes.

## Introduction

Because laboratory testing has a serious impact on clinical decision making the main task of laboratory professionals is to ensure high quality of test results. Implementation of high-quality test tubes can assure reliable test results and optimize laboratory work-flow through reducing the need for blood specimen re-collection as well as shortening time needed for sample preparation. Introducing new test tubes into routine application requires their prior verification (*1*). The procedure is well defined in Clinical and Laboratory Standards Institute (CLSI) guideline GP-34A (*2*).

For a long-time serum was considered the standard sample for analysis in clinical chemistry (*3*). In recent years plasma has become the preferable sample, especially for emergency laboratory analytes, because of time-saving advantages and lower risk of haemolysis (*3*-*5*).

BD Rapid Serum Tubes (RST) (Becton, Dickinson and Company, Franklin Lakes, USA) are vacuum tubes containing a thrombin-based clot activator and polymer gel which provides rapid serum separation and makes them suitable for emergency analyses (*6*). In BD RST tubes, under centrifugation conditions, gel becomes liquid and moves between plasma and cells. A thick gel barrier starts forming within 2 minutes resulting in trapping cells at the bottom of the plasma compartment. However, those tubes are susceptible to latent clot formation when collected from patients receiving high doses of anticoagulants, consequently delaying in reporting patient results (*6*). In order to improve turnaround time (TAT) and specimen quality, in 2016 the same company offered Barricor tubes (Becton, Dickinson and Company, Franklin Lakes, USA). Instead of polymer gel, these tubes contain a mechanical, chemically inert separator, and the interior tube wall coated with lithium heparin anticoagulant. Both, the tube stopper and the mechanical separator are lubricated with a silicone-based surfactant to facilitate product assembly. As the mechanical separator remains open during the entire centrifugation process, it enables efficient cell flow out of the plasma providing a much cleaner sample with less cell debris. According to the manufacturer, these tubes can withstand a relatively high centrifugal force (up to 5000xg), so as declared, a plasma sample can be obtained after a three-minute tube centrifugation at 4000xg ensuring a clean, high-quality sample, together with improved sample stability (*7*, *8*).

Serum and heparinized plasma specimens are considered to be equivalent for analysing many analytes, and it is not uncommon for hospital-based laboratories to receive plasma and serum specimens interchangeably (*6*). However, significant differences in the results obtained for some analytes are well documented (*6*, *9*).

The hypothesis of this study was that BD RST and BD Barricor tubes may be used interchangeably for analysing biochemistry and immunochemistry emergency laboratory panel parameters without changing the reference intervals, with the exception of potassium, as declared in manufacturer’s specification. Furthermore, we assumed that BD Barricor tubes ensure satisfactory plasma sample stability comparable to BD RST serum samples.

The aims of this study were: I) to evaluate if the analytes’ results provided by BD Barricor are comparable to the BD RST results and II) compare the sample stability difference, in both tubes at two time points (24 hours and 7 days), with the initial time.

## Materials and methods

### Subjects

Specimen collection was performed at the Osijek University Hospital from May to June 2017. Patients over 18 years old were randomly selected from different hospital departments (Table 1). Such a patient selection provided a real condition simulation and an adequate sample size for obtaining patients’ results with notably high and low values. Haemolysed or lipemic samples after centrifugation were considered as exclusion criteria. Finally, 52 patients with an average age of 63 (range from 20 to 87 years) were included, 27 males and 25 females. Any test result that was below the limit of detection (LoD) was excluded from further calculations.

**Table 1:** Patients included in the study

**Department**	**Female** **(N / total)**
Department of Nephrology	3 (6)
Department of Cardiovascular Disease	6 (12)
Department of Gastroenterology and Hepatology	3 (6)
Department of Endocrinology	3 (6)
Department of Nuclear Medicine and Radiation Protection	3 (5)
Department of Emergency Medicine	7 (17)

Informed consent was obtained from all patients enrolled in this study. The study was approved by the Ethics Committee and was in accordance with the Declaration of Helsinki.

### Blood sampling

Venipuncture was performed exclusively by one laboratory technician in the morning hours between 7.00 AM and 9.00 AM in the fasting state according to the proposed recommendations, except for patients admitted to the emergency department, where blood sampling was performed at the time of patient’s arrival but limited to the morning hours from 7.00 AM to 12.00 AM (*10*).

Blood sampling was performed randomly into two blood collection tubes: reference tube - BD RST (5 mL, Ref. 368774) and comparison tube - BD Barricor tube (4.5 mL, Ref. 65030). Immediately after blood collection, tubes were gently inverted, RST tube by five complete inversions and BD Barricor tube by eight complete inversions as recommended by the manufacturer. Tubes were transported in an upright position at room temperature to the emergency laboratory by the laboratory technician.

### Methods

Blood samples were centrifuged according to the manufacturer recommendation within 20 minutes from blood collection in a swing bucket centrifuge at room temperature: RST tubes for 10 minutes at 2000xg using Rotina 380R (Hettich, Tuttlingen, Germany) and Barricor tubes for 3 minutes at 4000xg using Allegra 2IR Centrifuge (Beckman Coulter, Brea, USA) (*8*). The RST centrifugation mode was in agreement with the daily routine in our laboratory. For each tube, following preanalytical requirements were monitored by the laboratory technician, according to the recommendations: incorrect filling of collection tubes (not within ± 10% of the stated draw volume), barrier formation, haemolysis and fibrin mass or fibrin strand presence (*1*). These characteristics were monitored after placing both the reference and the comparison tube in an upright position side by side.

Primary tubes were used for measuring most of the analytes, while aliquots were used for troponin I (TnI), myoglobin, thyroid stimulating hormone (TSH), free triiodothyronine (fT3) and free thyroxine (fT4) measurements. Sample aliquoting was performed immediately after centrifugation for the purpose of initial time sample stability measurement. Likewise, aliquots were taken again from the primary tubes at two time points for the purpose of sample retesting, following the predefined storage periods (24 hours and 7 days). The following analytes were measured in primary tubes using Beckman Coulter AU 480 analyser (Beckman Coulter, Brea, USA) with reagents from the same company, according to the manufacturer’s specification: sodium (Na), potassium (K), calcium (Ca), chloride (Cl), urea, creatinine, glucose, total protein, albumin, aspartate aminotransferase (AST), alanine aminotransferase (ALT), alkaline phosphatase (ALP), lactate dehydrogenase (LD), bilirubin, creatine kinase (CK), creatine kinase isoenzyme MB (CK-MB), amylase, gamma-glutamyltransferase (GGT), uric acid and C-reactive protein (CRP). All the above-mentioned analytes except CRP and electrolytes were measured with spectrophotometric methods. C - reactive protein was determined with an immunoturbidimetric method and electrolytes (Na, K, Cl) were determined by indirect potentiometry. All samples were also tested for lipemia/turbidity, icterus and haemolysis (LIH) interferences on the same analyser. Troponin I and myoglobin were measured using Dimension EXL with LM analyser (Siemens Healthcare Diagnostics, Newark, USA) based on a luminescent oxygen channelling assay (LOCI) technology. Finally, TSH, fT3 and fT4 were measured using ARCHITECT i2000SR (Abbott Diagnostics, Lake Forest, USA) analyser with a chemiluminescent microparticle immunoassay (CMIA) method. All measurements were carried out following our laboratory quality assurance procedures. Once the tests were performed, only primary tubes were stored at 4 °C. All analytes were retested at two time points (24 hours and 7 days) in the same way as previously described. Analytical performance specifications for tested parameters were all based on biological variation (*11*, *12*).

### Statistical analysis

Statistical analysis was performed using MedCalc for Windows, version 12.4.0.0. (MedCalcSoftware, Mariakerke, Belgium). The normality of distribution of investigated parameters was tested using the Kolmogorov-Smirnov test. Results are presented in accordance to their distribution: as average values and standard deviations (SD) for normally distributed parameters or median values and interquartile ranges (IQR) for non-normally distributed parameters. A paired t-test or a Wilcoxon test were run to calculate the differences between measured parameters. P value less than 0.05 (P < 0.05) was considered statistically significant. The bias for each analyte for between-tube comparison was calculated according to the formula: [(comparison tube result – reference tube result) / reference tube result] x 100, where BD RST represents the reference tube and BD Barricor the comparison tube. Average of all calculated results differences between the reference and comparison tube for each analyte represents the mean bias. The mean bias was compared to the current desirable specifications for inaccuracy that were previously calculated according to the formula as follows: B < (CV_w_^2^+CV_b_^2^)^1/2^, where CV_w_ stands for intraindividual variation and CV_b_ for interindividual variation (*12*, *13*). Differences between all measured parameters over time in the same tube type were calculated as follows: [(time point X result – time point 0 result) / time point 0 result] x 100, where X stands for 24 hours or 7 days, respectively. The mean difference for each analyte in the same tube over time (24 hours or 7 days) was compared to the current specifications for desirable allowable imprecision that was previously calculated according to the formula as follows: I < 0.5CV_w_^2^ (*13*). Analytes’ CV_w_ and CV_b_ were taken from European Federation of Clinical Chemistry and Laboratory Medicine (EFLM) Biological Variation Database, and if the data was not listed there (Ca, CK-MB, uric acid, bilirubin, TnI and myoglobin), data was taken from the Westgard’s database (*11*, *13*). Results of the mean biases between tubes and mean differences over time were considered acceptable when the calculated mean bias or difference for comparison did not exceed the desirable specification for inaccuracy or the desirable specification for allowable imprecision (*11*, *12*). Accordingly, all measured differences that exceeded the above-mentioned specifications were considered unacceptable. Passing-Bablok regression was performed for the results comparison. Results were presented with the regression equation (y = a + bx) with the corresponding 95% confidence intervals (CI), where intercept (a) represents the constant and slope (b) the proportional difference. If the 95% CI for intercept includes value 0 there is no constant difference between the measurements. Respectively, if the 95% CI for slope includes value 1 there is no proportional difference between measurements.

## Results

Visual inspection of analysed tubes (N = 52 BD RST and N = 52 BD Barricor) showed that tubes were adequately filled to their acceptable volume with the barrier formation. Furthermore, all tubes contained no fibrin mass or fibrin strands. No visible haemolysis was observed, which was later confirmed through routinely assessment of LIH on AU 480 analyser.

A few observed results for CK-MB, TnI, TSH, fT3 and fT4 parameters were below the LoD and these results were not utilized in any further calculations. Therefore, statistical analysis for listed analytes was carried out with a smaller data set (CK-MB: N = 35; TnI: N = 23; TSH: N = 49; fT3: N = 45; fT4: N = 49).

Table 2 summarizes the baseline results for all analytes observed in both investigated tubes, the BD RST as a reference tube, and the BD Barricor as a comparison tube, along with the observed patients’ results for each analyte and a corresponding sample size. Although 12 analytes (K, Ca, Cl, CK, ALP, GGT, uric acid, total protein, CRP, TnI, TSH and fT3) showed a statistically significant difference between tested tubes, for most analytes the calculated bias was within the desirable specifications for inaccuracy based on biological variation. Unacceptable bias between investigated tubes were obtained for K (- 4.5%) and total protein (4.4%), where calculated bias exceeded the predefined value of desirable bias (current desirable specifications for inaccuracy) (1.5% for K and 1.3% for total protein). Passing-Bablok regression did not show significant differences for most of the analytes. However, a systematic difference was observed for ALP, while both systematic and proportional differences were observed for LD, GGT, total protein, albumin and fT4.

**Table 2:** Baseline time-point comparison of analytes collected in the BD RST tube and the BD Barricor tube

**Analyte,** **unit**	**N**	**Observed patient’s results range**	**BD RST**	**BD Barricor**	**P**	**Bias (%)**	**Desirable bias (%)**	**PB 95% CI slope**	**PB 95% CI intercept**
Sodium, mmol/L	52	128-148	137 ± 3	137 ± 3	0.280	0.07	0.30	1.00 to 1.00	0.00 to 0.00
Potassium, mmol/L	52	2.8-6.5	4.4 ± 0.6	4.2 ± 0.6	< 0.001	- 4.47	1.47	0.86 to 1.00	- 0.20 to 0.41
Calcium, mmol/L	52	1.86-2.54	2.22 ± 0.16	2.20 ± 0.15	0.001	- 0.58	0.82	0.87 to 1.00	- 0.01 to 0.27
Chloride, mmol/L	52	92-120	104 ± 5	103 ± 5	0.005	0.22	0.43	1.00 to 1.00	0.00 to 0.00
Glucose, mmol/L	52	2.8-30.7	6.7 (6.2-7.6)	6.9 (6.2-7.7)	1.000	0.41	2.39	0.97 to 1.00	0.00 to 0.25
CK, U/L	52	11-2574	89 (56-132)	90 (56-131)	0.003	0.89	8.90	0.99 to 1.00	- 1.00 to 0.17
CK-MB, U/L	35	10-374	18 (13-20)	16 (13-19)	0.784	- 1.95	7.80	1.00 to 1.01	- 0.94 to 0.00
AST, U/L	52	10-231	29 (20-36)	29 (20-36)	0.183	0.14	5.63	0.95 to 1.00	0.00-1.61
ALT, U/L	52	3-200	24 (19-30)	25 (20-28)	0.741	2.70	7.75	0.96 to 1.00	0.00 to 1.01
ALP, U/L	52	32-498	75 (65-84)	74 (65-82)	< 0.001	- 2.43	6.10	0.96 to 0.99	- 1.64 to 1.29
LD, U/L	52	100-1369	211 (173-256)	213 (188-262)	0.371	1.66	3.38	0.92 to 0.98	5.06 to 20.93
GGT, U/L	52	7-1070	31 (25-36)	30 (24-34)	< 0.001	- 3.21	10.56	0.97 to 0.98	- 0.61 to -0.13
Urea, mmol/L	52	2.0-30.9	7.2 (6.3-8.5)	7.1 (6.3-8.4)	0.930	0.11	6.15	1.00 to 1.01	- 0.05 to 1.25
Creatinine, μmol/L	52	44-552	89 (79-104)	91 (80-106)	0.070	1.03	3.75	0.99 to 1.01	- 0.49 to 2.12
Amylase, U/L	52	24-979	60 (51-71)	61 (51-71)	0.116	- 0.45	7.73	1.00 to 1.00	0.00 to 0.00
Uric acid, μmol/L	52	86-930	387 ± 163	388 ± 165	0.007	0.27	4.87	1.00 to 1.02	- 4.14 to 1.00
Total protein, g/L	52	48.8-82.8	66.4 ± 7.7	69.1 ± 7.2	< 0.001	4.39	1.32	0.88 to 0.97	4.60 to 10.88
Albumin, g/L	52	22.1-48.4	37.4 ± 6.7	37.4 ± 6.4	0.760	0.12	1.43	0.93 to 0.98	0.71 to 2.51
CRP, mg/L	52	0.3-182.4	6.6 (3.1-12-5)	6.6 (3.0-12.3)	< 0.001	0.19	23.47	0.98 to 1.00	0.00 to 0.07
Bilirubin, μmol/L	52	6-314	12 (10-14)	12 (10-14)	0.091	0.02	8.95	1.00 to 1.00	0.00 to 0.00
TnI, ng/L	23	18-49,918	2903 (158-5386)	2972 (172-5536)	0.022	4.10	16.32	1.00 to 1.03	0.00 to 0.02
Myoglobin, μg/L	52	14-855	61 (50-81)	60 (49-80)	0.641	- 0.22	8.20	0.99 to 1.02	- 1.40 to 0.43
TSH, mIU/L	49	0.07-56.8	1.71 (0.927-2.55)	1.70 (0.934-2.62)	0.038	2.26	8.91	1.00 to 1.02	0.00 to 1.03
fT3, pmol/L	45	2.42-21.15	3.90 (3.44-4.27)	3.84 (3.42-4.50)	0.034	1.63	4.39	0.99 to 1.11	- 0.39 to 0.10
fT4, pmol/L	49	7.29-45.34	13.40 (12.98-14.28)	13.40 (12.95-14.06)	0.366	- 0.27	3.56	1.01 to 1.07	- 1.12 to -0.22
Data are presented as mean ± standard deviation, and median (interquartile range). %Bias - difference between the compared tube results and the reference tube results. PB - Passing-Bablok. CI - confidence interval. CK - creatine kinase. CK-MB - creatine kinase isoenzyme MB. AST - aspartate aminotransferase. ALT - alanine aminotransferase. ALP - alkaline phosphatase. LD - lactate dehydrogenase. GGT – gamma-glutamyltransferase. CRP - C-reactive protein. TnI - troponin I. TSH - thyroid-stimulating hormone. fT3 - free triiodothyronine. fT4 - free thyroxine. P value < 0.05 was considered statistically significant.									

The concentration changes over time in both tubes after 24 hours and after 7 days were compared to the current specifications for desirable allowable imprecision, and are shown in Table 3 (after 24 hours) and Table 4 (after 7 days). Most of the tested analytes showed satisfactory stability during a 24-hour storage period and met the predefined criteria based on the current specifications for desirable allowable imprecision. Unacceptable differences were noted for glucose, AST and LD measured in plasma specimens (BD Barricor), as well as for fT3 measured in serum specimens (BD RST), showing an unacceptable concentration change after a 24-hour storage period (Table 3). Less satisfactory results were obtained examining analytes’ long-term stability. Unacceptable differences after 7-day storage were obtained for Na, K, Ca, CKMB, AST, LD and TnI in both samples, while for glucose, ALP and albumin in plasma samples only (Table 4). Analytical coefficients of variation of all investigated analytes are summarized in the Supplementary table.

**Table 3:** Analytes stability comparison after a 24-hour storage period collected in the BD RST tube and the BD Barricor tube

**Analyte, unit**	**N**	**BD RST 0’**	**BD RST 24 h**	**Imprecision (%)**	**BD Barricor 0**	**BD Barricor 24 h**	**Imprecision (%)**	**Desirable imprecision (%)**
Sodium, mmol/L	52	137 ± 3	137 ± 3	0.10	137 ± 3	137 ± 3	0.01	0.25
Potassium, mmol/L	52	4.4 ± 0.6	4.4 ± 0.7	1.74	4.2 ± 0.6	4.2 ± 0.6	1.76	2.05
Calcium, mmol/L	52	2.22 ± 0.16	2.22 ± 1.18	0.30	2.20 ± 0.15	2.21 ± 0.16	0.32	1.05
Chloride, mmol/L	52	104 ± 5	104 ± 5	- 0.05	103 ± 5	104 ± 5	- 0.24	0.55
Glucose, mmol/L	52	6.7 (6.2-7.6)	6.7 (6.2-7.6)	- 0.92	6.9 (6.2-7.7)	6.6 (5.9-7.3)	- 3.88	2.55
CK, U/L	52	89 (56-132)	87 (57-131)	0.06	90 (56-131)	86 (55-128)	- 2.94	8.00
CK-MB, U/L	35	18 (13-20)	15 (13-19)	- 7.73	16 (13-19)	11 (10-16)	4.26	9.90
AST, U/L	52	29 (20-36)	29 (21-36)	1.83	29 (20-36)	31 (22-37)	4.85	4.75
ALT, U/L	52	24 (19-30)	24 (19-30)	1.88	25 (20-28)	25 (19-30)	1.76	5.20
ALP, U/L	52	75 (65-84)	75 (65-84)	- 0.81	74 (65-82)	74 (62-79)	- 1.77	2.70
LD, U/L	52	211 (173-256)	210 (170-249)	- 1.25	213 (188-262)	223 (201-261)	2.89	2.60
GGT, U/L	52	31 (25-36)	31 (25-38)	1.23	30 (24-34)	30 (24-36)	0.80	4.40
Urea, mmol/L	52	7.2 (6.3-8.5)	7.3 (6.4-8.4)	0.58	7.1 (6.3-8.4)	7.2 (6.4-8.4)	0.13	6.95
Creatinine, μmol/L	52	89 (79-104)	92 (79-106)	1.22	91 (80-106)	92 (80-104)	0.11	2.25
Amylase, U/L	52	60 (51-71)	61 (51-71)	- 0.29	61 (51-71)	60 (51-70)	- 0.78	3.30
Uric acid, μmol/L	52	387 ± 163	387 ± 166	0.01	388 ± 165	388 ± 164	0.17	4.30
Total protein, g/L	52	66.4 ± 7.7	66.5 ± 7.7	0.20	69.1 ± 7.2	69.0 ± 6.8	- 0.14	1.30
Albumin, g/L	52	37.4 ± 6.7	37.2 ± 6.7	- 0.49	37.4 ± 6.4	37.2 ± 6.4	- 0.48	1.30
CRP, mg/L	52	6.6 (3.1-12-5)	6.5 (3.0-12.6)	- 0.39	6.6 (3.0-12.3)	6.3 (3.1-12.5)	- 0.90	16.75
Bilirubin, μmol/L	52	12 (10-14)	12 (10-14)	- 3.40	12 (10-14)	12 (10-14)	- 2.66	10.90
**Analyte, unit**	**N**	**BD RST 0’**	**BD RST 24 h**	**Imprecision (%)**	**BD Barricor 0**	**BD Barricor 24 h**	**Imprecision (%)**	**Desirable imprecision (%)**
TnI, ng/L	23	2903 (158-5386)	2433 (157-5820)	- 2.71	2972 (172-5536)	2790 (172-5793)	- 0.44	7.03
Myoglobin, μg/L	52	61 (50-81)	60 (48-79)	- 0.59	60 (49-80)	58 (47-79)	- 0.88	7.00
TSH, mIU/L	49	1.71 (0.927-2.55)	1.70 (0.873-2.61)	- 2.10	1.70 (0.934-2.62)	1.69 (0.922-2.63)	0.60	7.95
fT3, pmol/L	45	3.90 (3.44-4.27)	3.96(3.49-4.46)	3.25	3.84 (3.42-4.50)	4.07 (3.51-4.55)	1.20	3.00
fT4, pmol/L	49	13.40 (12.98-14.28)	13.68 (13.09-14.15)	1.23	13.40 (12.95-14.06)	13.78 (12.76-14.42)	2.38	3.85
Data are presented as mean ± standard deviation, and median (interquartile range). PB - Passing-Bablok. CI - confidence interval. CK - creatine kinase. CK-MB - creatine kinase isoenzyme MB. AST - aspartate aminotransferase. ALT - alanine aminotransferase. ALP - alkaline phosphatase. LD - lactate dehydrogenase. GGT – gamma-glutamyltransferase. CRP - C-reactive protein. TnI - troponin I. TSH - thyroid-stimulating hormone. fT3 - free triiodothyronine. fT4 - free thyroxine.								

**Table 4:** Analytes stability comparison after a 7-day storage period collected in the BD RST tube and the BD Barricor tube

**Analyte, unit**	**N**	**BD RST 0**	**BD RST 7 days**	**Imprecision (%)**	**BD Barricor 0**	**BD Barricor 7 days**	**Imprecision** **(%)**	**Desirable imprecision (%)**
Sodium, mmol/L	52	137 ± 3	138 ± 3	0.50	137 ± 3	138 ± 4	0.30	0.25
Potassium, mmol/L	52	4.4 ± 0.6	4.6 ± 0.7	4.76	4.2 ± 0.6	4.4 ± 0.7	7.20	2.05
Calcium, mmol/L	52	2.22 ± 0.16	2.24 ± 0.17	1.16	2.20 ± 0.15	2.23 ± 0.15	1.11	1.05
Chloride, mmol/L	52	104 ± 5	104 ± 5	0.38	103 ± 5	104 ± 5	- 0.04	0.55
Glucose, mmol/L	52	6.7 (6.2-7.6)	6.7 (6.2-7.4)	- 1.39	6.9 (6.2-7.7)	5.8 (5.0-6.5)	- 19.3	2.55
CK, U/L	52	89 (56-132)	86 (56-136)	- 0.75	90 (56-131)	84 (56-136)	- 1.56	8.00
CK-MB, U/L	35	18 (13-20)	16 (12-19)	- 12.3	16 (13-19)	13 (12-18)	- 17.9	9.90
AST, U/L	52	29 (20-36)	30 (23-37)	5.22	29 (20-36)	33 (26-39)	13.2	4.75
ALT, U/L	52	24 (19-30)	24 (18-27)	- 4.64	25 (20-28)	24 (19-30)	- 1.83	5.20
ALP, U/L	52	75 (65-84)	76 (63-81)	- 0.96	74 (65-82)	68 (59-75)	- 7.37	2.70
LD, U/L	52	211 (173-256)	188 (158-239)	- 8.86	213 (188-262)	278 (234-299)	25.6	2.60
GGT, U/L	52	31 (25-36)	32 (25-38)	0.88	30 (24-34)	31 (25-41)	3.90	4.40
Urea, mmol/L	52	7.2 (6.3-8.5)	7.3 (6.4-8.4)	2.13	7.1 (6.3-8.4)	7.3 (6.4-8.6)	1.67	6.95
Creatinine, μmol/L	52	89 (79-104)	88 (80-103)	0.34	91 (80-106)	88 (80-103)	1.12	2.25
Amylase, U/L	52	60 (51-71)	60 (52-71)	- 0.56	61 (51-71)	60 (53-70)	- 0.48	3.30
Uric acid, μmol/L	52	387 ± 163	359 (327-435)	0.81	388 ± 165	357 (328-424)	0.13	4.30
**Analyte, unit**	**N**	**BD RST 0**	**BD RST 7 days**	**Imprecision (%)**	**BD Barricor 0**	**BD Barricor 7 days**	**Imprecision** **(%)**	**Desirable imprecision (%)**
Total protein, g/L	52	66.4 ± 7.7	66.1 ± 7.5	- 0.20	69.1 ± 7.2	69 ± 7.1	- 0.58	1.30
Albumin, g/L	52	37.4 ± 6.7	37.2 ± 6.8	- 0.55	37.4±6.4	36.5 ± 7.5	- 1.99	1.30
CRP, mg/L	52	6.6 (3.1-12-5)	6.1 (3.0-13.3)	- 0.62	6.6 (3.0-12.3)	6.1 (3.0-13.3)	- 0.98	16.75
Bilirubin, μmol/L	52	12 (10-14)	11 (10-13)	- 7.60	12 (10-14)	11 (10-13)	- 6.69	10.90
TnI, ng/L	23	2903 (158-5386)	2056 (202-5974)	- 19.4	2972 (172-5536)	2364 (200-5736)	- 15.2	7.03
Myoglobin, μg/L	52	61 (50-81)	57 (45-83)	- 2.35	60 (49-80)	59 (45-81)	- 2.71	7.00
TSH, mIU/L	49	1.71 (0.927-2.55)	1.65 (0.826-2.52)	0.47	1.70 (0.934-2.62)	1.75 (0.881-2.56)	0.62	7.95
fT3, pmol/L	45	3.90 (3.44-4.27)	3.78 (3.63-3.93)	1.95	3.84 (3.42-4.50)	3.75 (3.43-4.13)	- 1.83	3.00
fT4, pmol/L	49	13.40 (12.98-14.28)	13.18 (13.04-13.81)	0.28	13.40 (12.95-14.06)	13.69 (13.13-13.99)	1.46	3.85
Data are presented as mean ± standard deviation, and median (interquartile range). PB - Passing-Bablok. CI - confidence interval. CK - creatine kinase. CK-MB - creatine kinase isoenzyme MB. AST - aspartate aminotransferase. ALT - alanine aminotransferase. ALP - alkaline phosphatase. LD - lactate dehydrogenase. GGT – gamma-glutamyltransferase. CRP - C-reactive protein. TnI - troponin I. TSH - thyroid-stimulating hormone. fT3 - free triiodothyronine. fT4 - free thyroxine.								

## Discussion

This study evaluated BD Barricor tubes in relation to BD RST tubes in terms of test results comparison and analyte stability. Our results indicate that BD Barricor tube represents an acceptable alternative for BD RST tubes, except for measuring K and total protein. Analyte stability over a 24-hour storage period was acceptable for most analytes in both tubes, except for glucose, AST and LD in BD Barricor tubes and for fT3 in BD RST tubes. Stability of 15 analytes (Cl, CK, ALT, GGT, urea, creatinine, amylase, uric acid, total protein, CRP, bilirubin, myoglobin, TSH, fT3 and fT4), over a 7-day storage period, was acceptable in both tubes, while three analytes (glucose, albumin and ALP) showed better 7-day stability in BD RST tubes.

In recent years, only few BD Barricor tube evaluations were performed, mostly encompassing samples from healthy participants (*4*, *7*, *13*-*15*). Nevertheless, to the best of our knowledge, this is the first study evaluating BD Barricor tubes in relation to BD RST tubes in terms of analytes’ comparability and long-term stability of 25 analytes from in-patient samples. Such approach provided real hospital conditions ensuring a broad measuring range and finally obtaining a reliable bias and imprecision results.

Analyte results comparison given from both tubes were in line with our expectations and in agreement with previous examinations comparing serum and plasma specimen results (*9*, *14*-*16*). Kosem *et al.* compared eight biochemistry tests from BD RST and BD Barricor tubes with the BD Serum Separator Tubes (SST) as reference tubes (*16*). Their study was performed in haemodialysis patients. By comparing BD SST with BD Barricor tubes, they obtained an unacceptable bias for K and AST. Another recently published study by Arslan *et al.* along with K, reported an unacceptable bias for AST, LD and glucose in BD Barricor compared to serum samples (*9*). Difference in K concentration between plasma and serum tubes was observed in all mentioned studies (bias ranged from - 4.9 up to - 7.1%) and it was similar to the findings in our study (*9*, *14*-*16*). Our study, together with all mentioned studies, found a higher bias for K than the one stated for desirability in both the EFLM Biological Variation Database or Westgard’s database (*11*, *12*). In literature, higher K concentration in serum when compared to plasma, is mostly attributed to the release of K from platelets during platelet aggregation and degranulation in the clotting process, and this can probably be attributed to our study.

Positive bias for total protein in BD Barricor was also in accordance with other studies’ reports where bias ranged from 3.9 to 5.2% and could be explained with the presence of fibrin and fibrinogen in plasma samples that are also measured in the spectrophotometric reaction for total protein (*6*, *9*).

Unacceptable differences after a 24-hour storage period, in comparison to the baseline measurements, were observed for glucose, AST and LD in BD Barricor, and for fFT3 in RST tubes. The findings in our research are consistent with other studies data. Demeester *et al.* investigated the stability of 21 routine chemistry parameter in both S-Monovette lithium heparin and BD Barricor sample tubes at 5 different time points through 7 days (*17*). Imprecisions observed for glucose and AST after a 24-hour storage period were - 4.4% and 4.1%, respectively. Finally, these authors did not find unacceptable changes for LD (1.1%) after a 24-hour storage period. According to their data, acceptable glucose analysis postponement, in both investigated plasma tubes, was only 6 hours. This was also in accordance with the results presented in the Brandhorst *et al.* study, where authors investigated the stability of different clinical chemistry parameters in four different lithium heparin plasma tubes (*18*). Their data show that the acceptable glucose analysis postponement was to be between 9 and 15 hours, depending on the tube used. Dupuy *et al.* investigated the stability of nine analytes in BD lithium heparin tubes and BD Barricor tubes and they observed an AST activity increase after a 24-hour storage period in both BD lithium heparin (4.4%) and BD Barricor (5.6%) tubes, which is consistent with our results (*19*).

After a 7-day storage period unacceptable differences in comparison to the baseline measurements were observed for Na, K, Ca, CK-MB, AST, LD and TnI in both tubes, while for glucose, ALP and albumin in BD Barricor samples only, what is shown to be consistent with the results of other studies. Demeester *et al.* reported similar results after a 7-day storage period in BD Barricor plasma tubes for albumin, ALP, AST, glucose, LD, Na and K (*17*). Shimizu *et al.* obtained results comparable to ours for AST and LDH in serum samples after storing samples for 7 days on 4 °C (*20*).

As described in literature, serum displays a better sample stability compared to plasma (*4*). This is mostly attributed to blood cells remaining in plasma in higher concentration than in serum. Since AST and LD are present in cells, and glucose is a substrate of many cellular enzymes, the above-mentioned time-dependent stability point of issue may be caused by incomplete blood cell separation during the centrifugation process and subsequent blood cell lysis and releasing of intracellular content during the storage periods. This assumption is partially supported in the Dimeski *et al.* study, where authors demonstrated that BD Barricor tubes had a higher white blood cell count and markedly higher platelet count compared to the RST tubes (*21*). These findings could also explain higher AST and LD activities and lower glucose concentration in BD Barricor when compared to serum samples, as reported by the Arslan *et al.* study (*9*).

Passing-Bablok regression revealed no significant difference between results for majority of analytes. Proportional differences obtained for some analytes (ALP, LD, GGT, total protein, albumin and fT4) were small in magnitude (slope ranged from 0.93 to 1.04), and could not result in the concentration difference higher than the desirable specification for total allowable error according to the Westgard’s database, with the exception of total protein. As for systematic differences, the ones obtained for GGT, albumin and even fT4 (intercept ranged from - 0.74 to 1.59) were practically negligible. Significant systematic differences obtained for LD and total protein could be attributed to the already explained mechanisms (release of intracellular enzymes from residual blood cells during centrifugation for LD and presence of fibrin and fibrinogen for total protein).

Differences in fT3 concentration, after a 24-hour storage period, in RST tube is difficult to comment because we could not find other studies that were assessing fT3 stability in serum samples. Also, there is no consistency in fT3 stability at the two observed time points, because calculated imprecision after 7 days was found to be lower than the calculated imprecision after a 24-hour period. Besides, the desirable imprecision value, namely 3.0%, was calculated from the EFLM Biological Variation Database, while the desirable imprecision for FT3 in the Westgard’s database is found to be 4.0%.

It should be noted that our research has some limitations. Insufficient sample size for TnI due to the large proportion of patients with values below the LoD is one of the main shortcomings of our study. Additional limitation is the sample stability measurement at only two time-points. If we had comprised more time points in our study, we would be able to estimate time-dependent analyte stability more accurately. It should also be emphasized that the initial tubes draw volumes were not the same (5 mL for RST and 4.5 mL for Barricor). Also, we are aware that BD RST can be centrifuged at 4000xg for 3 minutes, same as BD Barricor (*8*). Although it would be better to make a comparison under the same centrifugation conditions, we opted for the centrifugation conditions of BD RST tubes according to our previous routine protocols. Still, since the minimum recommended clotting time for BD RST is 5 minutes, BD Barricor can contribute to improving laboratory performance by 5 minutes considering it can be centrifuged immediately.

In conclusion, BD Barricor tubes showed comparable results for almost all analytes, when compared to BD RST tubes, with the exception of K and total protein. Hence reference intervals for K and total protein should be adapted according to the sample type. All other analytes can be measured interchangeably, applying the same reference intervals. For most of the examined analytes, sample re-analysis can be performed in both tubes after 24 hours. If necessary, re-analysis can be performed in BD Barricor tubes after 7 days for most of the analytes, although BD RST tubes show better 7-day analytes stability over BD Barricor tubes. An advantage of BD Barricor tubes over BD RST tubes is an improved laboratory workflow due to the possibility of immediate centrifugation.
